# Biologically constrained optimization based cell membrane segmentation in *C. elegans* embryos

**DOI:** 10.1186/s12859-017-1717-6

**Published:** 2017-06-19

**Authors:** Yusuke Azuma, Shuichi Onami

**Affiliations:** Laboratory for Developmental Dynamics, RIKEN Quantitative Biology Center, 2-2-3 Minatojima-minamimachi, Chuo-ku, Kobe, Hyogo 650-0047 Japan

**Keywords:** Bioimage informatics, Cell membrane segmentation, Image processing, *C. elegans*, Embryonic development

## Abstract

**Background:**

Recent advances in bioimaging and automated analysis methods have enabled the large-scale systematic analysis of cellular dynamics during the embryonic development of *Caenorhabditis elegans*. Most of these analyses have focused on cell lineage tracing rather than cell shape dynamics. Cell shape analysis requires cell membrane segmentation, which is challenging because of insufficient resolution and image quality. This problem is currently solved by complicated segmentation methods requiring laborious and time consuming parameter adjustments.

**Results:**

Our new framework BCOMS (Biologically Constrained Optimization based cell Membrane Segmentation) automates the extraction of the cell shape of *C. elegans* embryos. Both the segmentation and evaluation processes are automated. To automate the evaluation, we solve an optimization problem under biological constraints. The performance of BCOMS was validated against a manually created ground truth of the 24-cell stage embryo. The average deviation of 25 cell shape features was 5.6%. The deviation was mainly caused by membranes parallel to the focal planes, which either contact the surfaces of adjacent cells or make no contact with other cells. Because segmentation of these membranes was difficult even by manual inspection, the automated segmentation was sufficiently accurate for cell shape analysis. As the number of manually created ground truths is necessarily limited, we compared the segmentation results between two adjacent time points. Across all cells and all cell cycles, the average deviation of the 25 cell shape features was 4.3%, smaller than that between the automated segmentation result and ground truth.

**Conclusions:**

BCOMS automated the accurate extraction of cell shapes in developing *C. elegans* embryos. By replacing image processing parameters with easily adjustable biological constraints, BCOMS provides a user-friendly framework. The framework is also applicable to other model organisms. Creating the biological constraints is a critical step requiring collaboration between an experimentalist and a software developer.

**Electronic supplementary material:**

The online version of this article (doi:10.1186/s12859-017-1717-6) contains supplementary material, which is available to authorized users.

## Background

Owing to recent advances in microscopic technology, biological labeling and digitization technology, large-scale live imaging with high spatiotemporal resolution is now possible. The live imaging technique accompanied by advanced computational methods has realized the automated analysis of biological dynamics. The usual automated technique, cell tracking during embryonic development, has been successfully performed in the nematode *Caenorhabditis elegans* [1–4], zebrafish [5], and *Drosophila melanogaster* [6, 7].

The embryonic development of *C. elegans* proceeds through a stereotypical pattern of cell divisions, known as an invariant cell lineage. Automated cell tracking exploits the invariant cell lineage, enabling large-scale systematic analysis of cellular dynamics in wild type and mutant embryos. Comparing the cell tracking results of different individuals, researchers have revealed small differences in cell division timings, cell cycle lengths and cell positions during embryonic development [1, 3, 8]. Cell tracking has been combined with the reporter gene expression of multiple genes and merged onto a reference lineage for gene expression profiling [9, 10]. Noting that the generated profile distinguishes nearly all pairs of embryonic cells [10], Du et al. [11] constructed a strategy that detects homeotic transformations on genetic perturbations by comparing the mutant and wild-type profiles, and infers the system-level mechanistic model of differentiation [11].

On the other hand, cells form stereotypical shapes during essential cell movements [12], cell–cell interactions [13] and morphological changes [14, 15]. Such shape changes have mainly been analyzed by visual inspection, which limits the analysis to only part of the embryonic development [16]. Given that automated cell tracking enables a wide variety of systematic analyses, its application to cell shape dynamics would provide us with unprecedented knowledge.

In cell tracking analysis, fluorescently labeled cell nuclei are segmented by image processing and are temporally associated [17]. The cell shape dynamics can be captured by a nearly identical procedure, which labels the cell membranes rather than the nuclei. However, membrane segmentation is more difficult than nuclear segmentation, not only because the segmentation itself is more complex, but also because a high segmentation quality is required.

Whereas cell nuclei are thick, well-separated spherical structures, cell membranes are thin planar shapes that contact each other, forming complicated networks. The segmentation is especially difficult for membranes that are parallel to the focal planes. In conventional confocal microscopy, the effective sampling resolution depends on the point spread function, which is worse in the axial (*z*) direction than in the planer (*x–y*) direction. Therefore, membranes parallel to the focal planes are sometimes imaged discontinuously. Additionally, unlike nuclei (which remain spherical), spherical cells can become squashed or expand lamellipodia and filopodia. These dynamics are difficult to segment using a shape model.

The primary objective of nuclear segmentation is to quantify the cell positions, so the segmentation quality is less important for nuclear shapes than for cell shapes. In membrane segmentation, the analysis must quantify the cell shape features, so the segmentation quality is critical.

High segmentation quality is not easily maintained, because the image quality (signal to noise ratio) is degraded at deeper focal planes by light scattering, absorption, and aberration [18], and at later time points by photo-bleaching [19]. As the fluorescence distribution in cell nuclei approximates a Gaussian distribution [1], a nucleus can be localized by detecting the locally bright centric region. However, when extracting cell shape features, the dimmer regions must be detected as well, which is very challenging.

Seeding the membrane segmentation with nuclei has successfully supported membrane segmentation [20–22], but is insufficient for accurate segmentation. Therefore, researchers have adopted additional sophisticated image processing techniques.

In the first step of sophisticated image processing, the membrane signals are enhanced by combining multiple filters. The filter sets include Gaussian or median filters and Hessian-based membrane enhancement filters [21, 23], rank filters and Difference of Gaussian filters [24], anisotropic filters and histogram equalization, and geodesic curvature filters and edge detectors [20]. Once the membrane signals are enhanced, the discontinuous or gap regions are filled by iterative morphological closing [21] or tensor voting [23]. Alternatively, a segmentation that is robust to gaps, such as active mesh framework [22] or viscous watershed [25], is applied.

In all approaches, the parameters must be adequately set to achieve sufficient performance. The parameters include the window size and sigma in filtering, the size of the morphological operations, the noise and watershed levels, the weights of the energy terms, the iteration number, and objective sizes such as cell diameter. Because each parameter must be optimally valued within a certain range, the number of parameter sets that must be tested increases in a combinatorial manner.

In the parameter adjustment process, the segmentation results must be evaluated in comparisons with the original membrane images. This evaluation is especially difficult for images in deeper focal planes, where the membrane signals are ambiguous. Because we must assume the cell shapes from upper focal planes, the evaluation in these deep planes is confounding and, of course, subjective. Additionally, a parameter set that properly segments the upper focal planes often fails in deeper planes, and vice versa. The same is true for images between early and late time points. Thus, we must seek a parameter set that performs accurate segmentation through all focal planes and time points, which is complicated and requires visual inspection.

When developing a new segmentation method or applying an existing method to a new problem, the parameter adjustments and segmentation assessments are iterated until the accuracy of the segmentation reaches the required level. As the number of parameter sets is enormous, and evaluating the segmentation results is complicated and reliant on human imagination, this iterative evaluation process is subjective, laborious and time-consuming.

In this study, we developed a new framework for cell shape extraction in *C. elegans* embryos, which automates both the segmentation and evaluation processes. Because it optimizes an objective function under biological knowledge-based constraints, our framework is named BCOMS (Biologically Constrained Optimization-based cell Membrane Segmentation). The performance of BCOMS is demonstrated in comparisons with cell shapes in a *C. elegans* embryo.

## Results

### Design

**Fig. 1:**
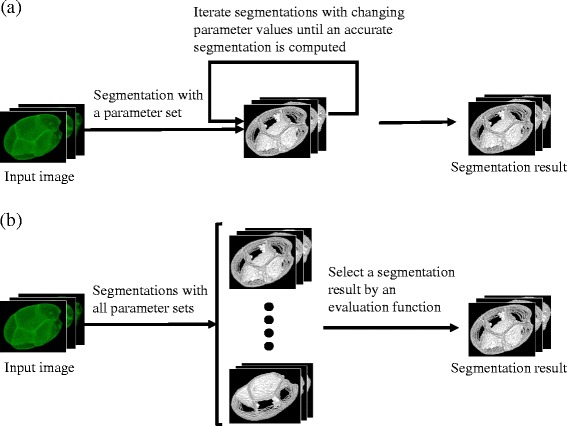
Comparison of typical and BCOMS approaches. Schematic comparison of membrane segmentation processes in typical (**a**) and BCOMS (**b**) approaches. **a** Typical approaches iterate segmentation of an input image with a parameter set, evaluation of the segmentation result, and adjustment of the parameter set until the segmentation is sufficiently accurate. **b** In BCOMS approach, segmentations are computed with all parameter sets and the optimal segmentation is selected by an evaluation function. The icons indicate the processed images and segmentation results, which are 3D time-lapse data

When segmenting cell membranes by a newly developed or existing method, we must adjust all parameters in the method for an accurate segmentation. Typical approaches iterate the following three steps: perform segmentation with a parameter set, evaluate the segmentation result, and adjust the parameter set. The iterations terminate when an accurate segmentation is computed (Fig. 1a). The main concept of the BCOMS framework is automation of this process (Fig. 1b). The segmentations in BCOMS are exhaustively performed over the whole parameter space and the optimal segmentation is selected by an automated evaluation method. The evaluation method is formulated as a constrained optimization problem:

1 $$ \max \mathrm{imize}\; f(x), x=\left({x}_1,{x}_2,\cdots, {x}_l\right) $$

2 $$ \mathrm{subject}\;\mathrm{to}\; g(x)={c}_e $$

3 $$ h(x)\overset{>}{=}{c}_u $$

where *f*(***x***) is the objective function to be maximized in the optimization problem. The vector ***x*** is the set of all parameter values included in the segmentation method, and *l* is the number of parameters in the method. *g*(***x***) and *h*(***x***) are given functions that satisfy the equality and inequality constraints (Eqs. (2) and 3), respectively). These functions ensure that the optimization problem is solved under biologically acceptable conditions. Therefore, the goal of this optimization problem is to find a set of parameter values ***x***_*o*_ that maximize the objective function *f*(***x***) under the equality and inequality constraints. The final segmentation result is the segmentation result calculated with the parameter set ***x***_*o.*_ The functions *f*, *g* and *h* are differently determined in each segmentation problem.

**Fig. 2:**
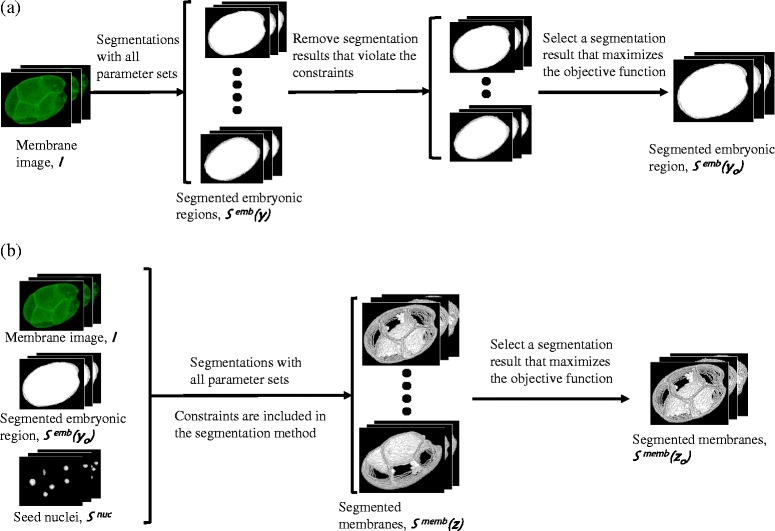
Schematic of the BCOMS framework. Schematic representation of embryonic region segmentation (**a**) and cell membrane segmentation (**b**) processes. In the two-step segmentation process of the cell membrane, the optimal segmentations are selected by solving optimization problems under defined constraints. The icons indicate the processed images and segmentation results, which are 3D time-lapse data. Names and variables of the icons (for example, *I*, *S* ^*emb*^ *(* ***y*** *)* and *S* ^*memb*^ *(* ***z*** *)*) are defined in the main text

The BCOMS realized the concept by a two-step segmentation framework; embryonic region segmentation using a level set method [26, 27], and cell membrane segmentation using a segmented nuclei-seeded watershed (Fig. 2, see Methods for details of the segmentation method). By segmenting the cell membrane, cellular regions are also simultaneously segmented. The nuclei-seeds can be prepared by two-color imaging, the cell membrane and cell nucleus are recorded in separate channels sequentially, and then segmenting the cell nuclear regions. The cell nucleus images are only used for giving the seeds.

In the embryonic region segmentation process (Fig. 2a), the segmentation results computed over the whole parameter space are evaluated by the following evaluation function:

4 $$ \max \mathrm{imize}\;\frac{\sum_p{I_p}^{\ast }{S}_p^{edge}\left(\boldsymbol{y}\right)}{\sum_p{S}_p^{edge}\left(\boldsymbol{y}\right)},\boldsymbol{y}=\left({y}_1,{y}_2,\cdots, {y}_m\right) $$

5 $$ \mathrm{subject}\;\mathrm{to}\kern0.24em \sum_p{S_p^{nuc}}^{\ast }{S}_p^{\overset{\sim }{emb}}\left(\boldsymbol{y}\right)=0 $$

6 $$ \frac{V\mathit{\min}\left(\boldsymbol{y}\right)}{V\mathit{\max}\left(\boldsymbol{y}\right)}\overset{>}{=}0.95 $$

Eq. (4) maximizes the objective functions, and Eqs. (5) and (6) define the equality and inequality constraints, respectively. The vector ***y*** is a set of all parameter values included in the embryonic region segmentation method, and *m* is the number of parameters in the method (the parameters are listed in Additional file 1: Table S1). *S* ^*emb*^(**y**) is the segmented embryonic region calculated with the parameter set ***y***. *S* ^*edge*^(**y**) is created by extracting an edge on each focal plane of the segmented embryonic region *S* ^*emb*^(**y**). *S*_*p*_^*edge*^(**y**). is the binary value of pixel *p* in *S* ^*edge*^(**y**). *I*_*p*_ is the intensity value of pixel *p* in membrane image *I*. The * operator denotes multiplication, namely, each pixel intensity value in the membrane image *I* is multiplied by the corresponding pixel’s binary value in *S* ^*edge*^(**y**). Σ_*p*_ denotes summation over the pixel indices *p*. Note that all images and segmentation results are 4D (3D time-lapse) data. Therefore, the objective function measures the average intensity value of the pixels that are segmented as the embryonic edge in the membrane image. *S*_*p*_^*emb*^(**y**) and *S*_*p*_^*nuc*^ denote the binary values of pixel *p* in the segmented embryonic region *S* ^*emb*^(**y**) and in the seed nuclei image, respectively. The seed nuclei image contains the segmented nuclear regions created by a previously developed image processing method (see Methods for details). The ~ operator represents inversion of a binary image. Therefore, the equality constraint (5) ensures that all nuclei are enclosed in the segmented embryonic region. *V*_*min*_(***y***) and *V*_*max*_(***y***) are the minimum and maximum volumes of the segmented embryos calculated with the parameter set ***y***, respectively. The volume is calculated by counting the pixels contained in each segmented embryonic region at each time point. The inequality constraint (6) ensures that the embryo maintains a near-stable volume throughout its development, as observed in our experiments. The inequality constraint excludes segmentations in which the later-stage embryonic regions are shrunken. Shrinking in later-stage embryos occurs by photo-bleaching, which degrades the image quality. To cancel this effect, our method adjusts the contraction bias by changing the weight parameters in the level set method according to the image brightness (see Methods for details). The inequality constraint ensures the correct performance of this adjustment.

**Fig. 3:**
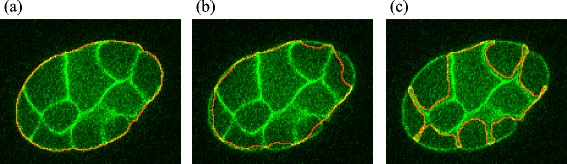
The objective function is maximized when the segmented membranes are just on the membranes. Embryonic region segmentations of a membrane image computed with different parameter sets. Red lines indicate the edges of the segmentation results. The value of the objective function is maximized when the edge locates just on the outermost membrane (**a**), and reduces when the edge locates inside this membrane (**b**). The value may again increase when the edge locates on the inner membranes (**c**). However this result is rejected because it violates the equality constraint (Eq. (5) that forces the embryonic regions to enclose all seed nuclei

The value of the objective function is maximized when the edge of the segmented embryonic regions locates on the outermost membranes (Fig. 3a), and decreases when the edge locates inside these membranes (Fig. 3b). When the edge locates along the inner membranes (Fig. 3c), the objective value may again increase but these segmentations are rejected by the equality constraint (5) that forces the embryonic regions to enclose all seed nuclei.

In the cell membrane segmentation process (Fig. 2b), the segmentation results computed over the whole parameter space are evaluated by the following evaluation function:

7 $$ \max \mathrm{imize}\;\frac{\sum_p{I_p}^{\ast }{S}_p^{memb}\left(\boldsymbol{Z}\right)}{\sum_p{S}_p^{memb}\left(\boldsymbol{Z}\right)},\boldsymbol{Z}=\left({Z}_1,{Z}_2,\cdots, {Z}_n\right) $$

where the vector ***Z*** is the set of all parameter values included in the cell membrane segmentation method and *n* is the number of parameters in the method (the parameters are listed in Additional file 1: Table S1). *S*_*p*_^*emb*^(***Z***) is the binary value of pixel *p* in the segmented cell membranes calculated with the parameter set ***Z***. No explicit constraints are given for the evaluation function (7). However, the watershed segmentation in the cell segmentation method is seeded with nuclei (see Methods for details), and each seed nucleus is enclosed by its self-formed region. By ensuring that each nuclear region is enclosed in its own cellular region, this method imposes an implicit biological constraint corresponding to Eq. (5) in the evaluation of embryonic region segmentation. An inequality constraint corresponding to Eq. (6) is not imposed, because the cell size may change.

### Comparison with ground truth

**Fig. 4:**
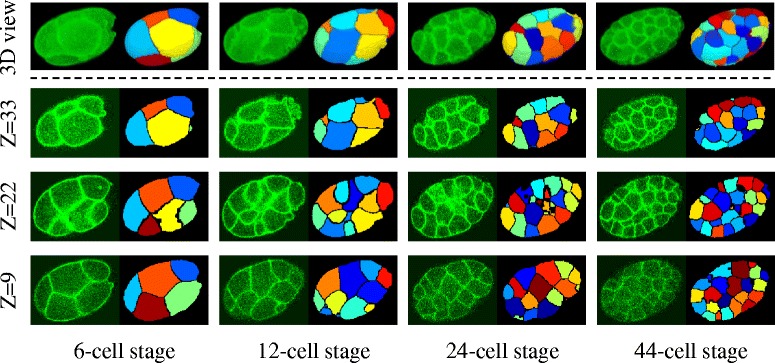
Accurate segmentations across focal planes and time points Shown are the membrane images and segmentation results computed by BCOMS at representative developmental stages and focal (*Z*) planes. The bottom and top focal planes are denoted as *Z* = 1 and *Z* = 36, respectively. The top panels are the 3D volume renderings of the membrane images and segmentation results. Cellular regions are rendered in different colors and the segmented membranes and background are rendered in black

To extract the cell shape dynamics, we recorded the embryonic development of *C. elegans* in the two- to 54-cell stages by 3D time-lapse imaging. For membrane segmentation by the BCOMS framework, we labeled the cell nucleus and cell membrane of the embryo as mCherry and GFP, respectively. Applying BCOMS to the image data, we acquired the segmentation results (Fig. 4, see also Additional file 2: Figure S1). The cell shapes were accurately segmented across all focal planes throughout the developmental period, including the deep focal planes (*z* = 9) where the image quality was reduced, and at later developmental stages. The segmentation results revealed a variety of cell shapes, from spherical to squashed.

As BCOMS was developed for automated extraction of the cell shape dynamics from the segmentation results, we must evaluate the accuracy of the extracted cell shapes. For this purpose, we created a ground truth of the 24-cell stage embryo by manual segmentation. This stage was selected because the cells contact each other in all three dimensions, and are sufficiently large to evaluate their shape features. Gastrulation in *C. elegans* is initiated around the 26-cell stage, so accurate segmentation of the embryo at this stage is essential for analyzing the cell shape dynamics during morphogenesis.

To quantitatively measure the cell shapes, we selected 25 cell shape features that are widely used in the image processing field. These features include volume, perimeter length, centroid location, cell length, surface area, convexity and sphericity. We computed the shape features of every cell in both the BCOMS result and the ground truth, then calculated their deviations by subtracting the feature values of the segmentation result from those of the ground truth, and dividing the absolute values of the results by the ground truth values. The average deviation among all features was 5.6% ± 3.5% (mean ± SD; the results of 13 representative features are shown in Table 1, and the results of all features are shown in Additional file 3: Table S2).

**Table 1:** Deviations between BCOMS and ground truth for 13 representative cell shape features

	Deviation (%)
Volume	3.4
PerimeterXY	3.3
PerimeterYZ	9.1
PerimeterZX	10.5
CentroidX	0.8
CentroidY	0.9
CentroidZ	0.9
Width	4.6
Height	7.3
Depth	9.4
Surface area	7.4
Convexity	3.3
Sphericity	5.3

Currently, no method can automate the extraction of cell shapes in *C. elegans* embryos. Therefore, we compared our result with the cell shapes of *Drosophila* and mouse embryos extracted by RACE software [21]. Stegmaier et al. [21] calculated the deviations of 22 cell shape features and averaged them over both organisms, obtaining 9.8% ± 4.9%. As the 25 features used in our comparison include all 22 of their features, we recalculated the average deviation of 22 shape features previously calculated by BCOMS, and obtained 5.6% ± 3.7%. Although the evaluated organisms are different, this comparison confirms that our segmentation result is sufficiently accurate to analyze cell shape features.

### Analysis of deviation

The deviations are expected to be larger in the *z* direction (where the resolution is poorer) than in the *x–y* direction. To confirm this expectation, we compared the deviations of the cell lengths in the *x*, *y* and *z* directions. Indeed, the deviation was larger along the *z* axis (9.4%) than along the planar directions (4.6% and 7.3% in the *x* and *y* directions, respectively). Likewise, the perimeter lengths deviated more on the YZ and ZX planes (9.1% and 10.5% respectively) than on the XY planes (3.3%). These results support the larger deviation in the *z* direction than in the *x–y* direction.

**Fig. 5:**
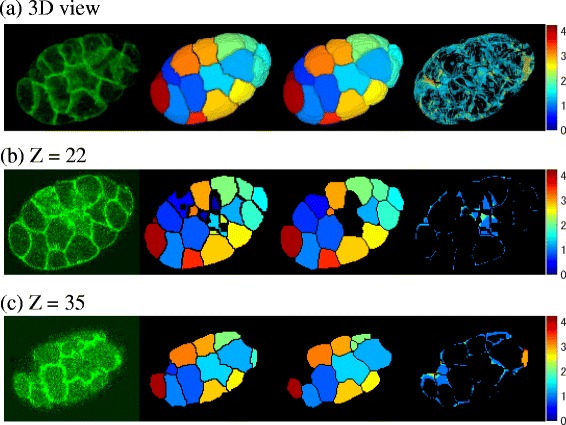
Differences between automated segmentations and ground truth caused by membranes parallel to the focal planes. (*Left* to *right*) Original image, BCOMS, ground truth, and pixel-level difference in the 24-cell stage embryo. 3D view images (**a**) and 2D view images (**b**, Z=22; **c**, Z=35). The cellular regions in the BCOMS segmentations and the ground truth are represented by different colors. For each pixel in each cell of the automated segmentation result, the pixel level difference was measured as the distance from the nearest pixel in the corresponding cell of the ground truth, and is displayed in pseudo color. The differences increase at the contacting surfaces of adjacent cells (**b**), and at bare surfaces that do not contact with other cells (**c**)

To further analyze the cause of the deviation, we measured the distance of each pixel in each cell of the automated segmentation result from the nearest pixel in the corresponding cell of the ground truth (right images of Fig. 5, see also Additional file 4: Figure S2). In most of the cells, the edges differed by approximately one pixel from the ground truth. As the drawn line cannot pass through the exact center of the membrane, some deviation is inevitable. Moreover, the computationally segmented membranes are one pixel thick and are three-dimensionally connected (in a 26-connected neighborhood). On the other hand, manual segmentation cannot be drawn to pixel-level accuracy. Considering these difficulties, deviations of a few pixels are acceptable.

The differences were larger in some locations. Representative focal planes with deviations exceeding two pixels are shown in Fig. 5, b and c. Regions of large difference include the contacting surfaces of adjacent cells (Fig. 5b) or bare surfaces that do not contact with other cells (Fig. 5c). In both scenarios, the surfaces were parallel to the focal planes. In the contacting surfaces of adjacent cells (Fig. 5b), membrane signals were difficult to distinguish from noise even by manual inspection. These membranes are diagonal in the focal planes. Therefore, to accurately connect them between adjacent focal planes, we must imagine their three-dimensional shapes. Manual segmentation of these membranes is inherently less accurate than segmentation of membranes perpendicular to the focal planes. In addition, during watershed segmentation, a region of a cell might extend beyond the separating membrane with insufficient fluorescence signals. On the other hand, in bare surfaces that do not contact with other cells (Fig. 5c), the automated segmentation extracted the outer (dimmer) bare surfaces instead of the correct bare surfaces. Parameter sets that do not extract the dimmer bare surfaces cannot avoid the incursion of extracted bare surfaces inside the true embryonic region during the level set segmentation. Segmentations using these parameter sets were generated in the segmentation process but rejected by the constraints, which force the embryonic region to enclose all of the segmented nuclei. However, extraction of the dimmer bare surfaces is not necessarily inaccurate because these surfaces are brighter than the background. We conclude that the deviations between the BCOMS segmentations and the ground truth arise from extremely ambiguous membranes.

### Comparison between adjacent time points

As only a limited number of ground truths can be created by manual effort, we compared the segmentation results between two adjacent time points. Although the cell shapes may change throughout the cell cycle, they should be almost stable between two adjacent time points, because the time interval of our imaging is 15 s (1.1% of the mean cell-cycle period). We computed the shape features throughout the development of each cell, then calculated their deviations between two adjacent time points. The deviation was calculated by subtracting the feature values at the previous time-point from those at the later time point, and dividing the absolute values of the results by the feature values at the previous time point. Across all cell cycles and all cells, the average deviation among all features was 4.3% ± 2.7% (the results of 13 representative features are shown in Table 2, and the results of all features are shown in Additional file 5: Table S3). The average deviation was smaller than that between the automated segmentation result and the ground truth. This result suggests that BCOMS successfully segments the membrane throughout the early embryonic development of *C. elegans*.

**Table 2:** Deviations between two adjacent time points for 13 representative cell shape features

	Deviation (%)
Volume	2.5
PerimeterXY	6.3
PerimeterYZ	6.4
PerimeterZX	7.3
CentroidX	0.7
CentroidY	0.7
CentroidZ	1.1
Width	4.6
Height	4.1
Depth	4.2
Surface area	2.7
Convexity	4.7
Sphericity	1.6

## Discussion

### BCOMS framework

Our new framework BCOMS was developed for automated cell shape extraction in *C. elegans* embryos. BCOMS automates not only the segmentation process but also the evaluation process. The evaluation was automated by solving an optimization problem under biological constraints. To apply BCOMS, we must adjust the parameters to fit the biological constraints, rather than adjust the image processing parameters. This replacement of the parameter fitting provides obvious benefits to both users and developers, and can be regarded as the major contribution of BCOMS.

### Benefit to users

When solving new problems by existing segmentation methods, we must adjust the image processing parameters (noise threshold, window size and sigma value in filtering, and the weights of the energy terms). Such adjustment requires understanding of what is controlled by each parameter. Therefore, users need at least a basic knowledge of the underlying algorithm. To users unfamiliar with image processing, such as experimental biologists, gaining this understanding is non-trivial. BCOMS replaces the image processing parameters with biological constraints. In the present study, the constraints enclose the nuclei within the embryonic or cellular regions, and limit the volume change of the embryo or each cell to a certain range. The former constraint is parameter-free, and the parameter of the latter is easily understood by biologists. Therefore, this replacement converts the complicated image processing method to a more user-friendly framework.

### Benefit to developers

Although BCOMS was developed for cell membrane segmentation in *C. elegans*, this framework is applicable to membrane segmentation in other organisms. To this end, both the segmentation and evaluation processes can be customized. To customize the segmentation process, we can simply replace the segmentation method. For instance, our novel segmentation method can be replaced by an existing method. In the evaluation process, the objective function measures the consistency between the membrane image and segmentation results, and is applicable to membrane segmentation in other organisms. However, the biological constraints may need to be tailored to different organisms. For example, the constraint of embryonic size stability is not suitable for mouse embryos, because mouse embryos grow as their development proceeds. In such cases, the constraints must be newly constructed. If the constraints are too strict, no segmentations are generated. Conversely, if the constraints are too loose, biologically unacceptable segmentations are selected. As the number of constraints is unlimited, the segmentation should incorporate as many constraints as possible. These constraints can be constructed by users with no knowledge of image processing, but must be based on biological knowledge of the target organism. This will present no problem to researchers familiar with the target organism. To fully exploit the advantages of BCOMS, experimentalists should collaborate with software developers.

### Performance of BCOMS

The performance of BCOMS was validated by comparisons with the ground truth and by comparing the results at two adjacent time points. After analyzing the pixel-level deviation of the automated segmentation result from the ground truth in each cellular region, we found that the deviations increased on membranes parallel to the focal planes. In fact, these membranes were difficult to segregate even by manual methods. Therefore, even the ground truth is not necessarily accurate. Comparing the two segmentations, many of these difficult-to-separate membranes were more accurately segmented by BCOMS than by the manual method. Therefore, the accuracy of the automated segmentation is equivalent to that of manual analysis.

Additionally, the automated segmentation faithfully follows the segmentation “rule” that every cell is necessarily separated by membranes, and that the membranes are three-dimensionally connected into a 26-connected neighborhood. In contrast, adjacent cells in manual segmentation may be directly contacted with no intervening membranes. The faithful segmentation by the automated method is especially important in systematic large-scale analyses, where stereotypical data are desired.

### Future work

Here, the optimization problem was solved by exhaustive searching. Although the search was completed in reasonable computational time, the computational efficiency would be improved by applying a more sophisticated optimization method. In future work, we will explore various optimization methods, and identify the most suitable methods for our problem. To this end, we will clarify how each parameter relates to the objective function and the biological constraints. A more sophisticated computation will reduce the human labor and time consumption of BCOMS applications, achieving an efficient developmental environment and a user-friendly analysis framework.

## Conclusion

We developed a new framework BCOMS which automated extraction of cell shapes in developing *C. elegans* embryos. The accuracy of BCOMS was validated by comparisons with the ground truth and by comparing the results at two adjacent time points. By replacing image processing parameters with easily adjustable biological constraints, BCOMS provides a user-friendly framework. The framework is also applicable to other model organisms by customizing the biological constraints. This customization is a critical step requiring collaboration between an experimentalist and a software developer.

## Methods

### Sample preparation and image acquisition

The *C. elegans* strain was OD95 ltIs37 [(pAA64) pie-1p::mCherry::his-58 + unc-119(+)] IV. ltIs38 [pAA1; pie-1::GFP::PH(PLC1delta1) + unc-119(+)]. Worms were grown and maintained at 22 °C by a standard procedure [28]. The embryos were dissected from adult worms on glass slides and mounted in a solution of 20-μm beads in M9 buffer [29]. Cover slips were placed on the mounts and sealed with Vaseline. Time-lapse imaging was performed with a spinning disk confocal unit CSU-X1 (Yokogawa Electric Corp.) mounted on an Eclipse Ti-E microscope (Nikon Instruments Inc.) equipped with an EM-CCD camera (iXon DU-897, Andor Technology Ltd). Images were acquired using a CFI Plan Apo VC 60XWI objective lens and a piezo stage controller (Nano-Drive, Mad City Labs). For system control and image acquisition, we employed iQ (Andor Technology Ltd). The image resolution was 256 × 256 pixels (2× binning), the axial (*z*) resolution was 0.5 μm, and the time interval was 15 s. The focal planes and time points numbered 36 and 428, respectively.

### Two-step segmentation

We initially applied nuclei-seeded watershed segmentation with an additional seed located outside of the embryo, and defined the background. However, some cellular regions in the segmentation result were extended into the background and some of these extended cellular regions penetrated into other cellular regions through the background (Additional file 6: Figure S3). These discrepancies were caused by the insufficient fluorescent signals of the membranes located on the edge of the embryo, where the signal intensities are lower than those on the contacting surfaces of adjacent cells. To solve this problem, we replaced the background seed with separate segmentation of the embryonic region, before segmenting the cellular regions.

### Embryonic region segmentation

Segmentation of the embryonic region was performed by a level set method [26, 27], which achieves better smoothing properties of the resulting segmentation than the watershed method [30]. As the initial region for the level set method, we temporally summed the 4D (3D time-lapse) image to generate a 3D image stack, then extracted the pixels with intensities above the mean intensity.

In deeper focal planes, the images become dimmer and their quality degrades. To offset this degradation, we calculated the average intensity of the pixels enclosed by the initial contour on each focal plane of the 3D image stack, and multiplied it by an adjustment factor. At later time points, the images are similarly dimmed by photo-bleaching. To cancel this effect, we adjusted the contraction bias (corresponding to the weight of the curve-length term in [26]) in the level set segmentation according to the image brightness. Specifically, dimmer images were assigned a weaker bias than brighter images. The bias of each image stack at time *t* was calculated as

8 $$ cb(t)={c}^{\ast}\mathit{\operatorname{var}} s{(t)}^b. $$

Here, *vars(t)* is the intensity variance of the pixels enclosed by the initial embryonic region of the 3D image stack at time *t*, and *c* and *b* are parameters.

For embryonic region segmentation, the membrane images were first applied to a Gaussian filter, then processed by the level set method [26, 27] initialized by the pre-segmented contour.

### Cell nuclei segmentation

The nuclei were segmented by our previously developed scheme [31]. After computing the 3D Difference of Gaussian filtering, the method applies adaptive noise thresholding, followed by a size threshold that removes small regions. The segmentation in the developmental stages is almost error-free [31]. Here, the minimal errors in the segmentation results were removed by manual curation. Note that the segmentation was performed on cell nuclei images that are acquired in a different channel from that of the cell membrane images. The images are not shown in this manuscript.

### Cell membrane segmentation

Using the segmented nuclei as seeds for the watershed, we segmented the cellular regions. However, as the fluorescent signals on the membranes were insufficient, some of the cellular regions extended into adjacent cells beyond the separating membranes (see Additional file 7: Figure S4, the segmentation results with *α* = 0). To solve this problem, we created hybrid images *I* ^*hyb*^ by the following formula:

9 $$ {S}^{dist}= D\left[\overset{\sim }{S^{nuc}}\right] $$

10 $$ {}^{I_p^{hyb}}={I}_p+{\alpha}^{\ast }{S}_p^{dist} $$

where *S* ^*nuc*^ is the seed nuclei image. The ~ operator represents inversion of a binary image and *D*[*] denotes the Euclidean distance transform. *I*_*p*_ and *S*_*p*_^*dist*^ denote the intensity values of pixel *p* in the membrane image and in *S* ^*dist*^, respectively, and *α* is a parameter. The second term in Eq. (10) weights the intensities according to the distances of the pixels from the nuclei. As *α* increases, each region becomes increasingly less likely to extend far from the seed nucleus in the hybrid image *I* ^*hyb*^. For a given image, the number of parameters and the computational time are smaller in the watershed segmentation than in the level set. Therefore, the hybrid watershed method is appropriate for segmenting large-scale image data such as the image data in the present study.

The hybrid images were first input to an averaging filter. Next, the membranes were segmented by the nuclei-seeded watershed approach. The parameters used in this study are listed in Additional file 1: Table S1. The sample segmentation results and the values of the objective function computed with different *α*s are shown in Additional file 7: Figure S4. Cell membranes separating the cells were extracted as watershed lines with single-pixel thickness, and were three-dimensionally connected (in a 26-connected neighborhood). Therefore, the final cell membranes constituted the cell membranes separating the cells, and the surfaces of the embryonic region. Each of the extracted cellular regions was separated by segmented cell membranes.

### Creation of ground truth

The ground truth of the 24-cell stage embryo was manually created. First, the cellular regions were manually segmented on each image plane using the ROI manager in ImageJ (Rasband, W.S., ImageJ, U. S. National Institutes of Health, Bethesda, Maryland, USA, http://imagej.nih.gov/ij/, 1997–2016.). Next the segmented regions were associated with the image planes, reconstructing the 3D cells. In this reconstruction, performed by custom-made software, the overlapped regions of adjacent cells were equally divided.

## Additional files

Additional file 1: Table S1. Parameter values (XLSX 9 kb)

Additional file 2: Figure S1. Accurate segmentations across focal planes and time points, related to Fig. 4. Shown are the membrane images and segmentation results computed by BCOMS at representative developmental stages and focal (*Z*) planes. The bottom and top focal planes are denoted as *Z* = 1 and *Z* = 36, respectively. The top panels are the 3D volume renderings of the membrane images and segmentation results. The segmented membranes are rendered in green and background are rendered in black. Cellular regions are rendered in black in 2D view images and in dark blue in 3D view images. (PDF 222 kb)

Additional file 3: Table S2. Deviation between BCOMS and ground truth for all cell shape features (XLSX 9 kb)

Additional file 4: Figure S2. Differences between automated segmentations and ground truth caused by membranes parallel to the focal planes, related to Fig. 5. (Left to right) Original image, BCOMS, ground truth, and pixel-level difference in the 24-cell stage embryo. The segmented membranes are rendered in green in the BCOMS segmentations and the ground truth. Cellular regions are rendered in black in 2D view images (b, c) and in dark blue in 3D view images (a) and background are rendered in black. For each pixel in each cell of the automated segmentation result, the pixel level difference was measured as the distance from the nearest pixel in the corresponding cell of the ground truth, and is displayed in pseudo color. The differences increase at the contacting surfaces of adjacent cells (b), and at bare surfaces that do not contact with other cells (c). (PDF 96 kb)

Additional file 5: Table S3. Deviation between two adjacent time points for all cell shape features (XLSX 9 kb)

Additional file 6: Figure S3. Mis-segmentation of embryonic edge membranes. Original membrane images and watershed segmentation results at the 12-cell (a, *Z* = 8) and 44-cell (b, *Z* = 8) embryonic stages. Cellular regions are represented by different colors. The cellular regions colored in yellow extend into the background and penetrate into other cellular regions (white arrows) through the background. (PDF 47 kb)

Additional file 7: Figure S4. Differences of segmentation results on α values. Cell membrane segmentation results computed with different *α* values in Eq. (10). In these comparisons, the seeded watershed segmentation was not preceded by an average filter. Shown are representative results of 12-cell (a, *Z* = 9) and 44-cell (b, *Z* = 20) embryonic stages. (a, b) White arrows indicate cells that obviously extended into adjacent cells beyond the separating membranes. Cellular regions are represented by different colors. (c) Value of the objective function at each *α*, computed over the whole embryogenesis data (see segmentation result in the main text). (PDF 73 kb)

## Abbreviations

BCOMS: Biologically constrained optimization based cell membrane segmentation
*C. elegans*: *Caenorhabditis elegans*
