# Supplementary figures and images for: Biologically constrained optimization based cell membrane segmentation in C. elegans embryos
Source: BMC Bioinformatics. 2017 Jun 19;18:307. doi: 10.1186/s12859-017-1717-6 (PMC5477254; doi:10.1186/s12859-017-1717-6)

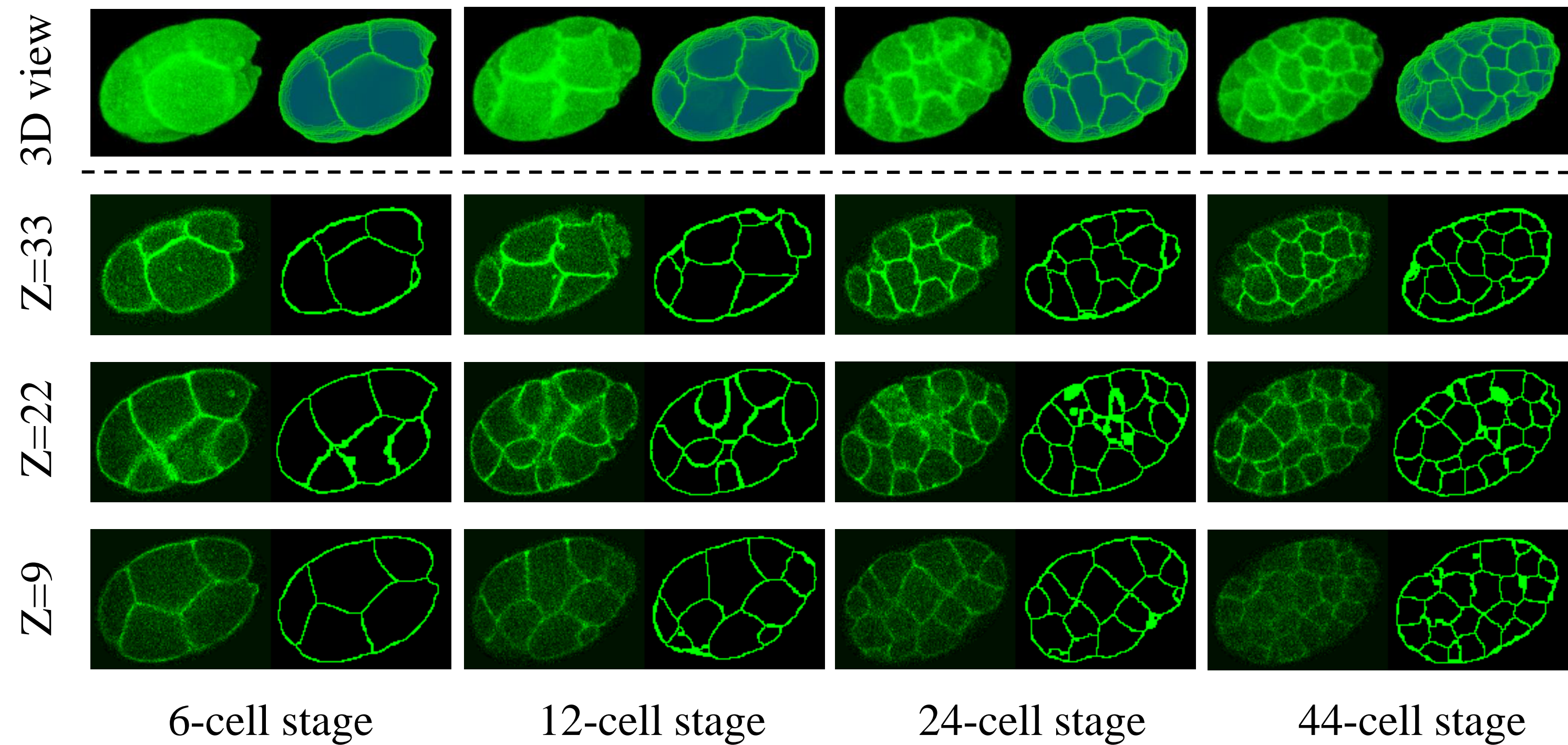

Supplement: Supplementary file 2 — Accurate segmentations across focal planes and time points, related to Fig. 4. Shown are the membrane images and segmentation results computed by BCOMS at representative developmental stages and focal (Z) planes. The bottom and top focal planes are denoted as Z = 1 and Z = 36, respectively. The top panels are the 3D volume renderings of the membrane images and segmentation results. The segmented membranes are rendered in green and background are rendered in black. Cellular regions are rendered in black in 2D view images and in dark blue in 3D view images. (PDF 222 kb) [file 12859_2017_1717_MOESM2_ESM.pdf]

(a) 3D view

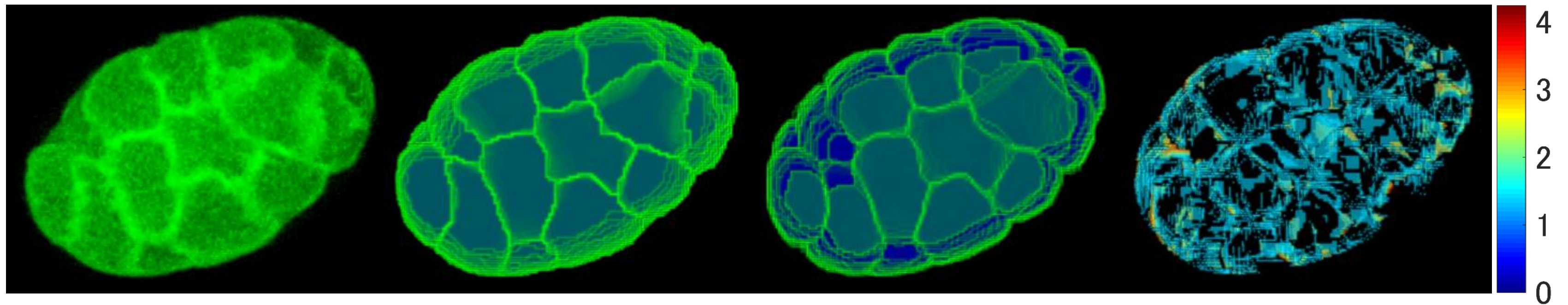

(b)  $Z = 22$

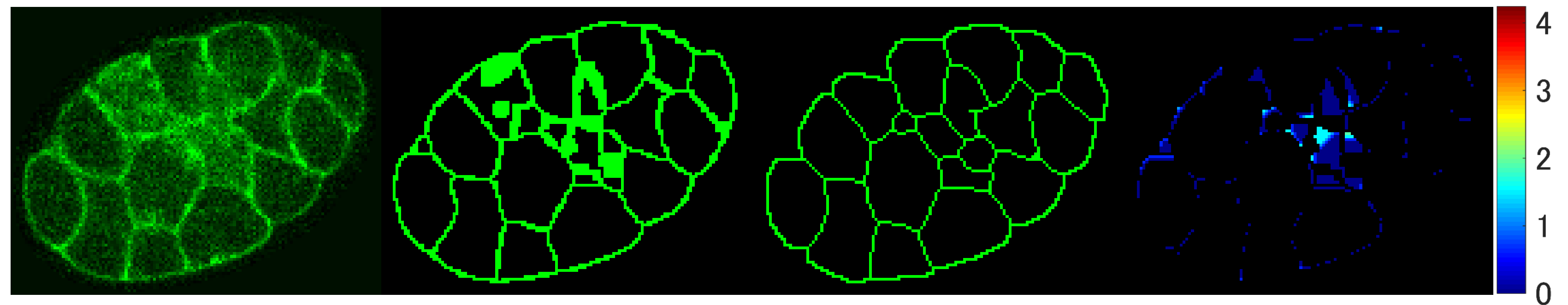

(c)  $Z = 35$

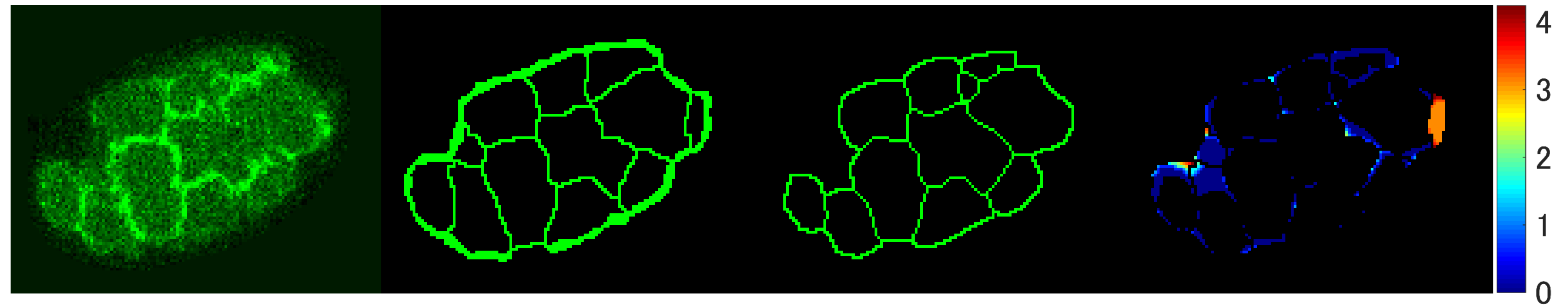

Supplement: Supplementary file 4 — Differences between automated segmentations and ground truth caused by membranes parallel to the focal planes, related to Fig. 5. (Left to right) Original image, BCOMS, ground truth, and pixel-level difference in the 24-cell stage embryo. The segmented membranes are rendered in green in the BCOMS segmentations and the ground truth. Cellular regions are rendered in black in 2D view images (b, c) and in dark blue in 3D view images (a) and background are rendered in black. For each pixel in each cell of the automated segmentation result, the pixel level difference was measured as the distance from the nearest pixel in the corresponding cell of the ground truth, and is displayed in pseudo color. The differences increase at the contacting surfaces of adjacent cells (b), and at bare surfaces that do not contact with other cells (c). (PDF 96 kb) [file 12859_2017_1717_MOESM4_ESM.pdf]

(a) 12-cell stage

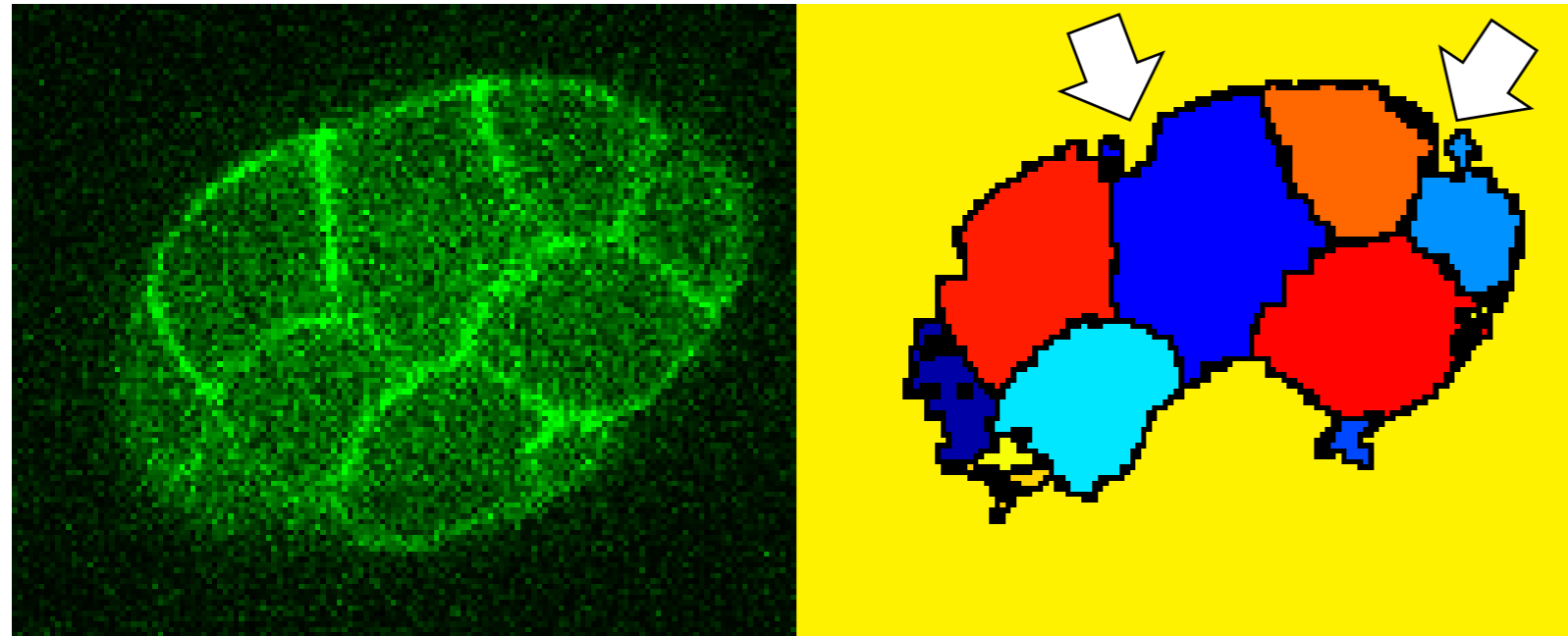

(b) 44-cell stage

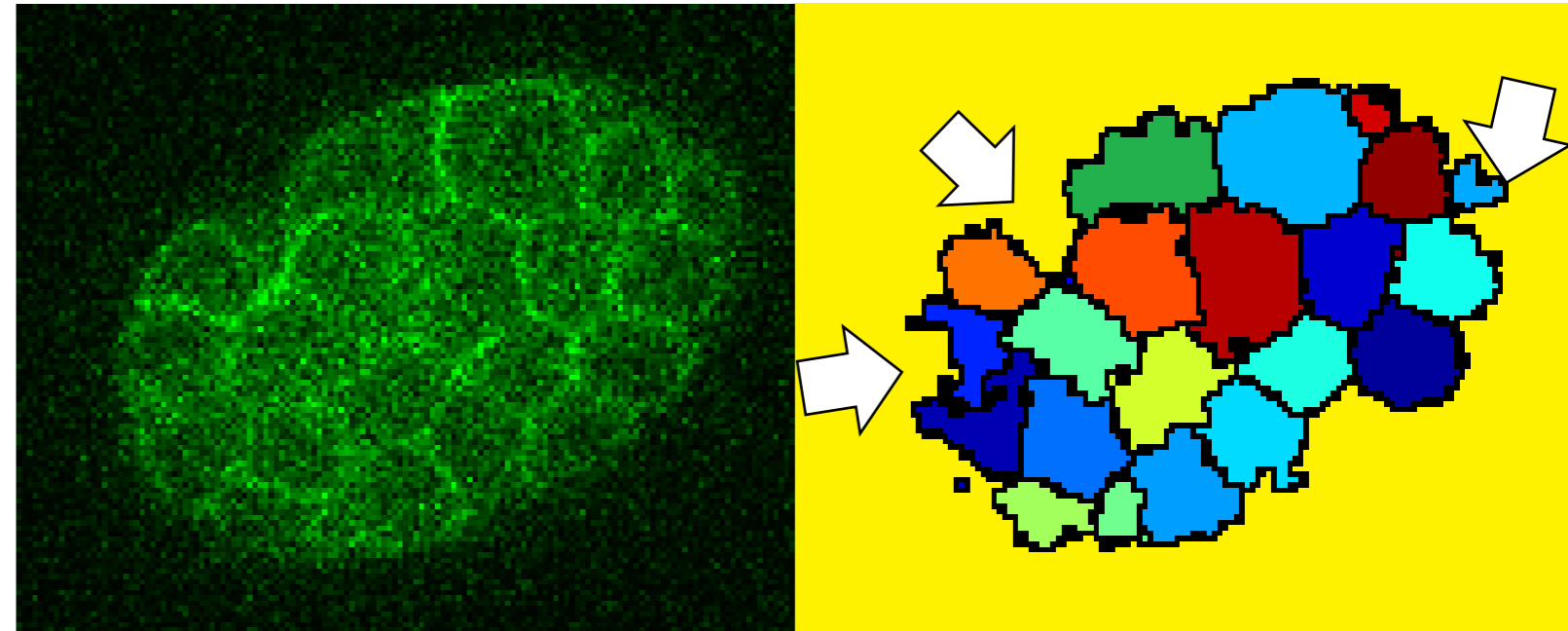

Supplement: Supplementary file 6 — Mis-segmentation of embryonic edge membranes. Original membrane images and watershed segmentation results at the 12-cell (a, Z = 8) and 44-cell (b, Z = 8) embryonic stages. Cellular regions are represented by different colors. The cellular regions colored in yellow extend into the background and penetrate into other cellular regions (white arrows) through the background. (PDF 47 kb) [file 12859_2017_1717_MOESM6_ESM.pdf]

(a) 12-cell stage

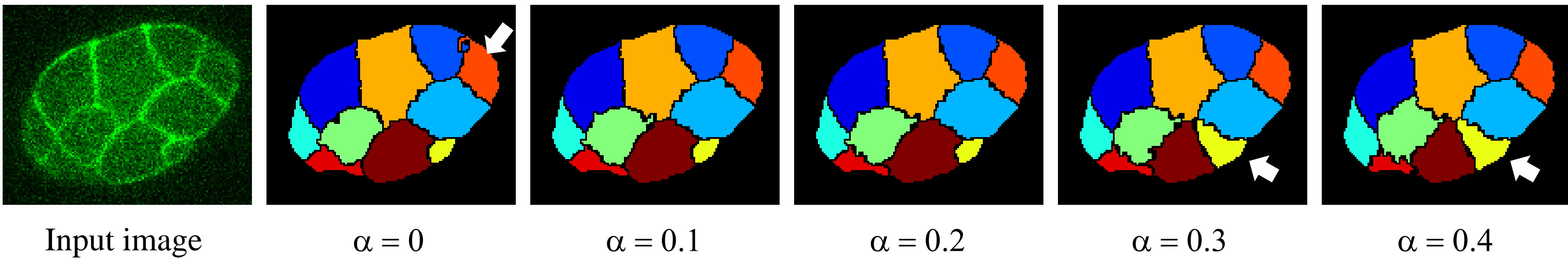

(b) 44-cell stage

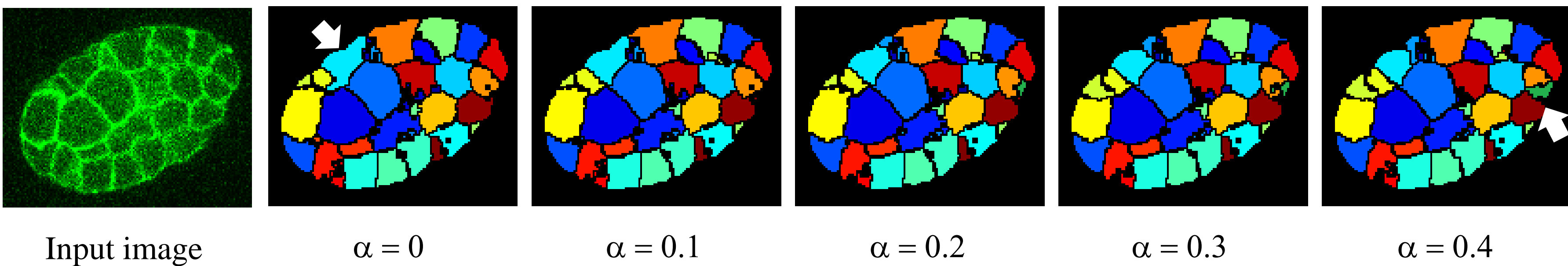

(c)

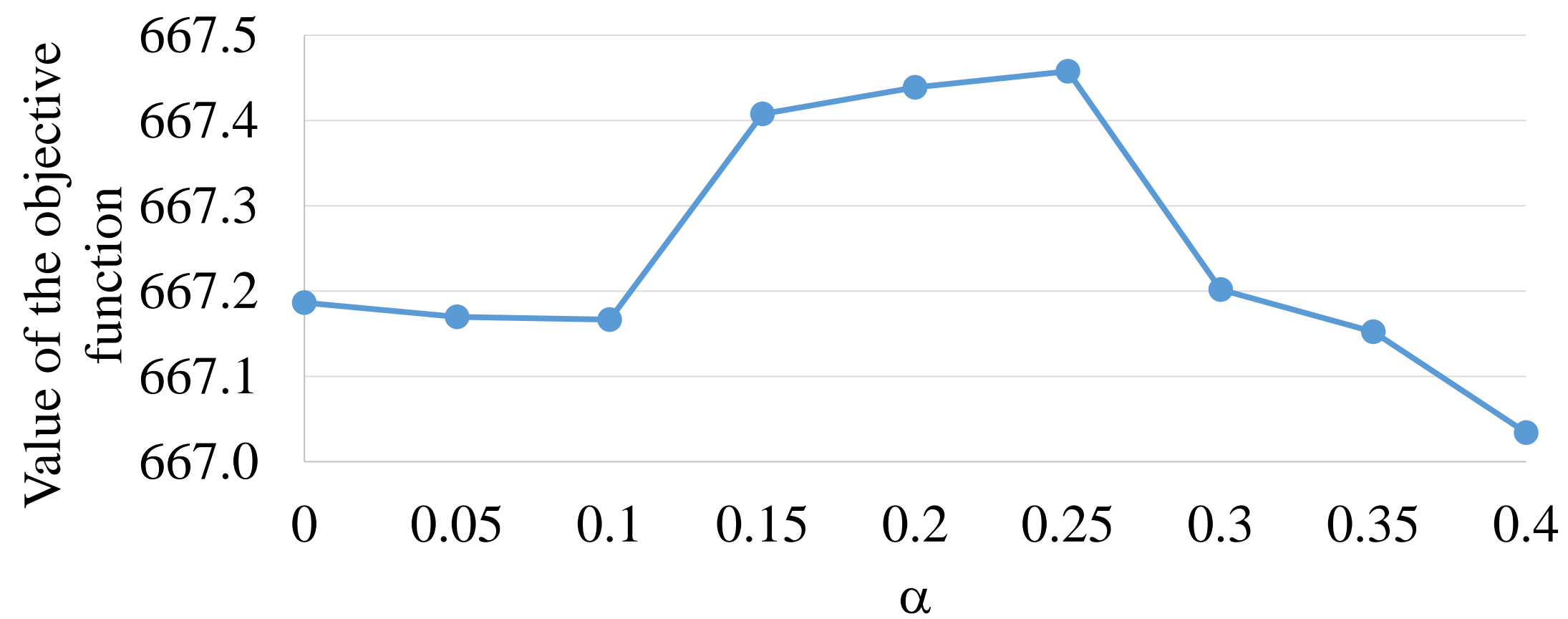

Supplement: Supplementary file 7 — Differences of segmentation results on α values. Cell membrane segmentation results computed with different α values in Eq. (10). In these comparisons, the seeded watershed segmentation was not preceded by an average filter. Shown are representative results of 12-cell (a, Z = 9) and 44-cell (b, Z = 20) embryonic stages. (a, b) White arrows indicate cells that obviously extended into adjacent cells beyond the separating membranes. Cellular regions are represented by different colors. (c) Value of the objective function at each α, computed over the whole embryogenesis data (see segmentation result in the main text). (PDF 73 kb) [file 12859_2017_1717_MOESM7_ESM.pdf]
